# Cretaceous sea turtle soft tissues clarify ancestry of scale loss in chelonioids

**DOI:** 10.1016/j.isci.2025.113641

**Published:** 2025-09-27

**Authors:** Benjamin P. Kear, Roy Nohra, Johan Lindgren, Márton Rabi, Mohamad Bazzi

**Affiliations:** 1 Department of Palaeobiology, Swedish Museum of Natural History, 104 05 Stockholm, Sweden; 2 Expo Hakel, Hâqel, Main Road, Byblos, Mount Lebanon 14014354, Lebanon; 3 Department of Geology, Lund University, Sölvegatan 12, 223 62 Lund, Sweden; 4 Department of Geosciences, University of Tübingen, Hölderlinstraße 12, 72074 Tübingen, Germany; 5 Department of Earth and Planetary Sciences, Stanford University, Stanford, CA 94305, USA

**Keywords:** Paleobiology, Paleontology, Zoology

## Abstract

Scale loss is a quintessential hydrodynamic adaptation in marine reptiles, and paralleled the pelagic specializations of Mesozoic ichthyosaurs, plesiosaurs, and metriorhynchid crocodylians, as well as the modern Leatherback Sea turtle (Dermochelyidae). By contrast, modern hard-shelled sea turtles (Cheloniidae) retain both scutes and scaly flippers, despite evolving from among partially scale-less antecedents after the earliest Eocene, ∼54 million years (Ma) ago. Here, we resolve the ambiguous ancestry of scale loss using the oldest known sea turtle (total-group Chelonioidea) soft tissues preserved in a mid-Cretaceous (middle-to-upper Cenomanian, ∼97 Ma) protostegid (basally divergent chelonioid) from Lebanon. This fossil combines scale-less flipper skin with a scuted carapace similar to other extinct chelonioids, but confirms lineage specific rather than ubiquitous scale loss in an ancestral states analysis. Scale-less skin is therefore an ancient sea turtle trait that was repeatedly modified from scaly ancestors within disparate chelonioid clades during their recurrent independent invasions of oceanic environments.

## Highlights

• Scale loss is a convergent trait linked to aquatic specialization in reptiles
• Some earliest sea turtles had both scale-less flippers and scuted shells
• Scale retention in modern sea turtles is ancestral and possibly habitat related
• Scale loss evolved repeatedly with oceanic lifestyles in sea turtles

## Graphical abstract

**Figure:**
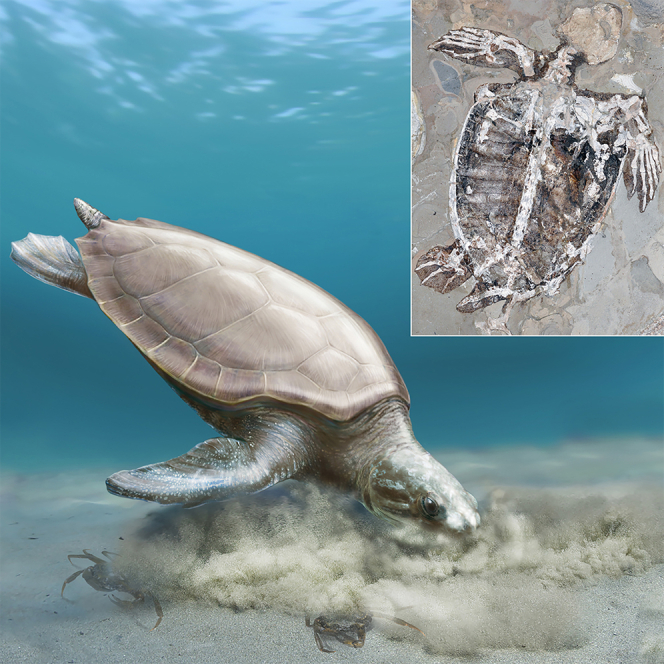

## Introduction

Chelonioids are the most highly specialized aquatic turtles, with an evolutionary history extending back to the Early Cretaceous, over ∼100 Ma.^1^ Modern chelonioids are represented by two seemingly closely related^2^^,^^3^^,^^4^ crown clades that are superficially distinguished by the presence or absence of epidermal scales. (1) Cheloniidae—incorporating the extant Loggerhead Sea turtle (*Caretta caretta*), Green Sea turtle (*Chelonia mydas*), Hawksbill Sea turtle (*Eretmochelys imbricata*), Ridley sea turtles (*Lepidochelys kempii*, *Lepidochelys olivacea*), and Flatback Sea turtle (*Natator depressus*), all of which retain an apparently ancestral condition^5^^,^^6^^,^^7^^,^^8^ of polygonal scales on the limbs and scutes (= mosaic shell scales^7^) on the carapace and plastron. (2) Dermochelyidae—which comprises a single extant species, the Leatherback Sea turtle (*Dermochelys coriacea*), characterized by smooth leathery skin covering the entire shell and body (at least in adults^9^). The adaptive tendency toward scale loss is also evident in habitually aquatic non-marine turtles (members of the clades Carettochelyidae and Trionychidae),^10^^,^^11^ and hallmarks the transition to pelagic lifestyles^12^ in advanced marine reptiles,^13^^,^^14^^,^^15^ with convergent mirroring by hair loss in obligate aquatic mammals (e.g., cetaceans).^12^

The presence of scutes in fossil turtles can be readily deduced from residual sulci delimiting their diagnostic outlines across the bony plates of the shell. Alternatively, the limb and body scales usually leave no skeletal traces (although scale-bearing limb osteoderms are known for some extinct terrestrial turtles^5^^,^^6^^,^^8^^,^^16^^,^^17^^,^^18^^,^^19^), and the preserved remnants of original soft tissues are exceptionally rare. The geologically oldest examples include limb scale impressions and phosphatized skin residues in Late Jurassic (upper Kimmeridgian and lower Tithonian platy limestone [“Plattenkalk”] deposits^3^ including the Altmühltal Formation^20^) thalassochelydian (Thalassochelydia) marine turtles consistent with *Thalassemys bruntrutana*,^3^ and the mouldic outline of a hindlimb paddle^21^ from the Early Cretaceous (upper Aptian, Crato Formation^22^) marine pan-pelomedusoid (stem-Pelomedusoides), *Araripemys barretoi*.^23^ In addition, scale-less skin has been surmised from the taphonomy and paleoenvironment of the mid-Eocene (lower Lutetian, Messel Formation^24^) non-marine carettochelyid, *Allaeochelys crassesculpta*.^25^

Among fossil sea turtles, “layers of black, probably carbonaceous material” were reported with a skeleton of the earliest Eocene (Ypresian, Fur Formation^26^) dermochelyid, *Eosphargis breineri* (Nielsen,^27^ p. 308). Shell osteoderms from another early dermochelyid, *Arabemys crassiscutata*,^28^ have been cited as timing chelonioid scute loss (or reduction) to the Paleocene (?Thanetian–Ypresian, Lina Member^29^^,^^30^^,^^31^), and perhaps prior to the group’s radiation into oceanic settings.^28^ Conversely, remains attributed to the earliest Eocene (Ypresian, Fur Formation^26^) crown-lineage chelonioids *Tasbacka danica*^32^ and *Eochelone*^10^^,^^33^ preserve shell scutes, but *Eochelone* also lacked flipper scales,^10^ possibly reflecting an independent invasion of neritic habitats.^10^ Tong et al.^34^ further documented soft tissues associated with an articulated skeleton (Mineral Museum Beirut [MIM] F50) of the protostegid *Rhinochelys nammourensis*. This specimen was excavated from the mid-Cretaceous (middle-to-upper Cenomanian) Nammoura Lagerstätte of the Sannine Formation,^35^ which is famous for producing animal and plant soft tissue traces, including reptilian scales.^36^ The Sannine Formation was laid down under shallow neritic conditions,^35^ and is today quarried for decorative building stone and commercially traded fossils in the valley of the Abraham River, ∼34 km along the 51M main coastal highway toward Jbeil (Byblos) from Beirut in Lebanon.^35^

*Rhinochelys nammourensis* belonged to a geologically early protostegid genus with a trans-oceanic distribution from the Northern European epicontinental Tethys to peri-equatorial eastern Gondwanan shelf during the late Albian-to-Cenomanian^2^^,^^34^^,^^37^ (after ∼105 Ma). The taxon is therefore pivotal for resolving the origins of scale loss in sea-going turtles. However, the initial description of MIM F50 (previously designated “ESC-2”^34^ in reference to the private collection of Francois Escuillié at *Eldonia* [https://www.spinosaure.com/]) only mentioned “weakly developed” carapace scute sulci (Tong et al.*,*^34^ p. 123) and “solid” fore- and hind flipper “skin impressions” (Tong et al.*,*^34^ p. 128), but did not discuss the soft tissue structures in any detail. We therefore used high-resolution cross-polarized (CP) and ultraviolet (UV) light photography to re-examine MIM F50 while on permanent public exhibition. Unfortunately, the specimen could not be removed from its mounting fixtures, and sampling for microscopic and spectroscopic analyses was not possible because the bones and soft tissues had all been coated with acrylic resin for stabilization.

## Results and discussion

### Description

**Figure 1:**
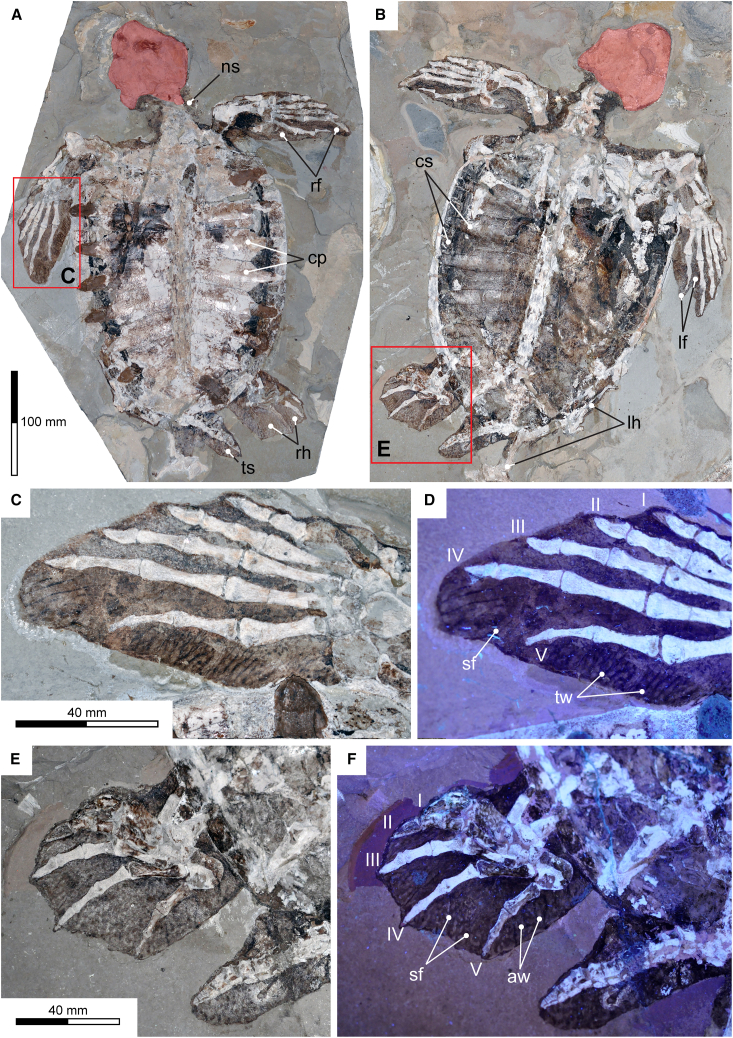
Oldest fossil sea turtle soft tissues (MIM F50) (A) Cross-polarized (CP) image of the part slab^34^ showing bony carapace in dorsal view (red fill = skull reconstruction). (B) CP image of the counterpart slab showing carapace scutes in internal view. (C) CP image of the left fore-flipper from (A). (D) Ultraviolet (UV) image of the left fore-flipper digits I–V and trailing edge from (A). (E) CP image of the right hind-flipper from (B). (F) UV image of the right hind-flipper digits I–V and webbed skin from (B). Anatomical abbreviations: aw, anastomosing wrinkles; cs, costal plates; cs, carapace scutes; lf, left fore-flipper skin traces; lh, left hindlimb elements; ns, neck skin traces; rf, right fore-flipper skin traces; rh, right hind-flipper skin traces; sf, skin folds; ts, tail skin traces; tw, trailing edge wrinkles. Scale bars = 100 mm in (A) and (B); 40 mm in (C)–(F).

MIM F50 consists of an articulated postcranial skeleton exposed on part and counterpart lithographic limestone^35^ slabs (Figures 1A and 1B). A crude skull reconstruction was made by commercial preparators prior to the Tong et al.^34^ study, and subsequent acquisition of both the part and hitherto undocumented counterpart slab by MIM. Comparisons between published low-resolution photographs^34^ and the current condition of MIM F50 indicate that no significant alterations were made to artificially enhance the skeletal or soft tissue remains for display. Nonetheless, the slabs were broken during quarrying and reassembled using colored glue. Some scute material was also stripped off by the commercial preparators to better expose the costal plates on the right side of the part slab. The costals have undergone minor restorations in plaster, and the missing proximal end of the right humerus was re-modelled on the counterpart slab. Lastly, the leading edges of the left fore-flipper and right hind flipper were outlined with tinted adhesive to increase their definition.

The preserved soft tissues of MIM F50 are still extensive, and include not only the carapace scutes on the counterpart slab, but also what we interpret as residual skin surrounding the bones of the left and right fore-flippers, right hind-flipper, tail, and neck. These remnant tissues form dark brown to black (presumably melanic) films that are identical to the diagenetically transformed and compacted organic integument identified in other fossil sea turtles.^10^^,^^32^^,^^33^ Indeed, they retain high-fidelity external features along the expanded trailing edge of the left fore-flipper on the part slab (Figures 1C and 1D), and between the splayed digits II–IV of the right hind-flipper on the counterpart slab (Figures 1E and 1F). These reveal a skin surface covered by anastomosing wrinkles that become more prominent and perpendicularly oriented against the flipper trailing edges. Interspersed deep folds likely represent postmortem distortion.^10^ Importantly, there is no evidence of the conspicuous polygonal scales found on the flippers of modern cheloniids,^10^ or on the paddle-like limbs of more basally divergent thalassochelydian marine turtles.^3^

**Figure 2:**
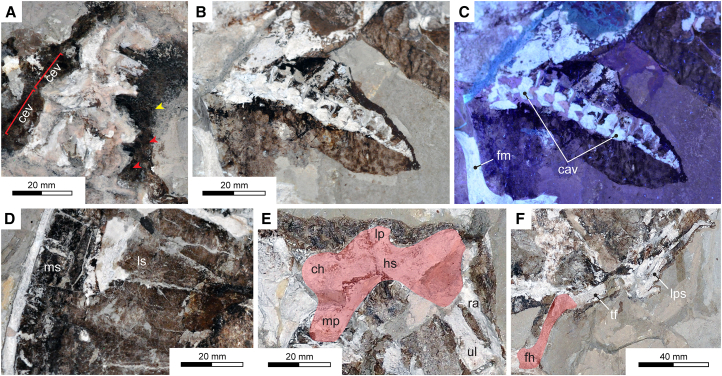
Oldest fossil sea turtle soft tissues (MIM F50) (A) Cross-polarized (CP) image of the cervical vertebrae with surrounding soft tissues showing possible skin folds (yellow arrow) and dark-colored spots (red arrows). (B) CP image of the caudal vertebrae and tail skin from the part slab (Figure 1A). (C) Ultraviolet (UV) image of (B). (D) CP image of the internal surface of the carapace scutes from the counterpart slab (Figure 1B). (E) CP image of the humerus (red fill) from the counterpart slab (Figure 1B). (F) CP image of the disarticulated distal hindlimb and femur (red fill) from the counterpart slab (Figure 1B). Anatomical abbreviations: cav, caudal vertebrae; cev, cervical vertebrae; ch, caput humeri; fh, femoral head; fm, femur; hs, humeral shaft; lp, lateral process; lps, left pedal elements; mp, medial process; ms, marginal scute; ra, radius; tf, tibia and fibula; ul, ulna; vs., vertebral scute. Scale bars = 20 mm in (A)–(E); 40 mm in (F).

Tong et al.^34^ (p. 115) considered MIM F50 to be an osteologically mature “adult” with “nearly complete carapace, forelimbs, partial hindlimbs, neck and tail, lacking skull.” Our personal observations concur with their skeletal assessment, although the right hindlimb is actually intact and articulated while the left hindlimb bones are disarticulated and dispersed behind the posterolateral margin of the carapace (best seen on the counterpart slab: Figure 1B). Tong et al.^34^ (p. 121) stated that the MIM F50 “carapace is exposed in ventral view”; but this is incorrect. Rather, eight pairs of costal plates (the last pair potentially meeting along the carapace midline) are exposed in dorsal aspect on the part slab (Figure 1A), while the neck and tail (Figures 2A–2C), together with right hindlimb and left fore-flipper (Figures 1C–1F) partly underlie the carapace (photographed by Tong et al.*,*^34^ p. 124, figure 6). The counterpart slab shows the internal surfaces of the vertebral and marginal scutes, which are in places covered by adhering patches of bone sheared off from the neural, costal, and peripheral plates when the limestone blocks were split (Figure 2D).

As noted by Tong et al.*,*^34^ the carapace of MIM F50 is longer than wide (270/212 mm in maximum preserved length/width: p. 115, Table 1) with a keeled midline (inferred from indentation of the neurals into the residual scute surfaces on the counterpart slab) and fontanelles between the costals and peripherals. It resembles those of other smaller-bodied protostegids, such as *Santanachelys*^39^ in possessing a smoothly rounded anterior peripheral margin. By contrast, the posterior-most peripherals and pygal are “undulated” or “serrated” (Tong et al.*,*^34^ p. 122), but differ from the distinctly scalloped peripherals found in *Calcarichelys*^40^ and possibly *Terlinguachelys*.^41^ Significantly, there is no evidence of skin contouring along the edges of the shell, which are otherwise surrounded by only a very thin dark outline delimiting the marginal scutes (Figure 1E).

**Table 1:** Bayesian ancestral states analysis using a total-group Chelonioidea phylogeny

Node	ANC State	Mean	Variance	L-HDP	U-HDP	Median	Min ESS	Avg ESS	PSRF
Total-group Chelonioidea	P(0) scales	0.93	0.01	0.76	1	0.96	306	684.48	1
	P(1) scale loss	0.07	0.01	0	0.24	0.04	306	684.48	1
Protostegidae + crown-group Chelonioidea	P(0) scales	0.71	0.01	0.50	0.87	0.71	326.27	330.77	1
	P(1) scale loss	0.29	0.01	0.13	0.50	0.29	326.27	330.77	1
Protostegidae	P(0) scales	0.66	0.01	0.47	0.87	0.66	210.36	352.67	1.01
	P(1) scale loss	0.34	0.01	0.15	0.53	0.34	210.36	352.67	1.01
*Rhinochelys*	P(0) scales	0.17	0	0.06	0.3	0.16	314.41	348.83	1
	P(1) scale loss	0.83	0	0.7	0.94	0.84	314.41	348.83	1
Ctenochelyidae + crown-group Chelonioidea	P(0) scales	0.6	0.01	0.39	0.81	0.6	233.31	279.54	1
	P(1) scale loss	0.4	0.01	0.19	0.6	0.4	233.31	279.54	1
Crown-group Dermochelyidae	P(0) scales	0.74	0.01	0.58	1	0.73	18.97	35.7	1.01
	P(1) scale loss	0.26	0.01	0	0.42	0.27	18.97	35.7	1.01
Crown-group Cheloniidae	P(0) scales	0.85	0.03	0.74	0.95	0.85	321.93	340.81	1
	P(1) scale loss	0.15	0.03	0.05	0.26	0.15	321.93	340.81	1

**Figure 3:**
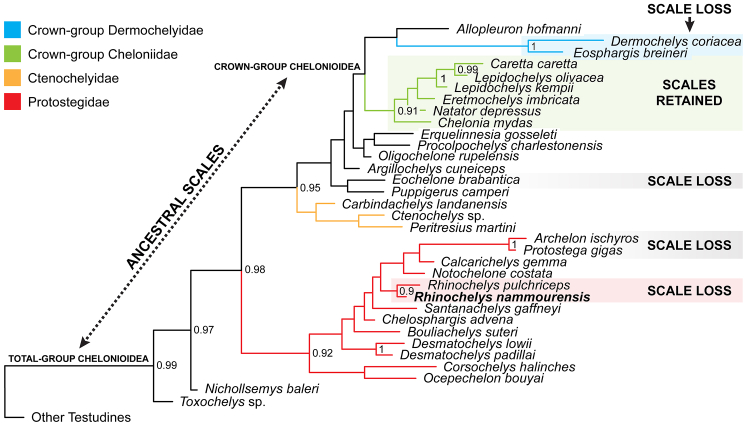
Bayesian phylogenetic context of ancestral scale loss in chelonioids Bayesian consensus tree from *MrBayes* v.3.2.7^38^ shows the phylogenetic placement of *Rhinochelys nammourensis* (MIM F50), the major clade nodes within total-group Chelonioidea (see Table 1), and posterior probability support values (PP) ≥0.9. Inferred instances of scale loss (see Table 1) occur in: *Rhinochelys* (red shading) and protostegids that lack scute sulci^47^^,^^49^ (gray shading); remains resembling the extinct cheloniid *Eochelone*^10^^,^^33^ (gray shading); and the extant dermochelyid lineage^9^^,^^10^^,^^11^ (blue shading). Scales are otherwise ancestral in chelonioids (dashed line) and retained in extant cheloniids (green shading).

Probability % (P) of the ancestral state (ANC State) being (0) = scales, versus (1) = scale loss at constrained target clade nodes calculated with *MrBayes* 3.2.7.^38^ Analysis parameters are explained in the STAR Methods. Tree topology is shown in Figure 3. Abbreviations: min ESS = minimum estimated sample size among runs; avg ESS = average estimated sample size among runs; L-HDP = lower 95% HDP interval; PSRF = Potential Scale Reduction Factor (1.0 = convergence between runs); U-HDP = upper 95% HDP interval.

Little can be discerned of the cervical vertebrae, but three or four are present anterior to the nuchal on the MIN F50 counterpart slab (Figure 2A). The surrounding soft tissue remnants are amorphous with dark-colored spots (perhaps original organic pigment patterning), and what may be skin folds visible around the base of the neck (Figure 2A). Tong et al.^34^ (p. 126) counted 14 articulated caudal vertebrae and mentioned that the tail “is long, extending well beyond the posterior margin of the carapace.” This proportionately resembles the male condition in sexually dimorphic living chelonioids,^42^ but the caudals of MIM F50 are compact and decrease in size distally (Figures 2B and 2C), which accords with the expected tail morphology of females.^43^ Irrespectively, we consider MIM F50 to be a male,^34^ with the females having comparatively shorter tails that were covered by the carapace (see Tong et al.*,*^34^ p. 116; Figure 1). As on the fore- and hindlimbs, soft tissue traces surround the caudal vertebrae and exhibit a similar surface texturing of anastomosing wrinkles and skin folds.

The humeri of MIM F50 are badly damaged. However, the outline of the left (=“right” of Tong et al.*,*^34^ p. 126) humerus is comparable to those tentatively associated with *Rhinochelys*.^2^ It also possesses a prominent lateral process that is much larger and more anteriorly projecting on the counterpart slab (Figure 2E) than that initially envisaged by Tong et al.^34^ based on the part slab. The medial process extends far proximally relative to the caput humeri^34^ (although depth of the intervening notch is uncertain), and the posterior profile along the humeral shaft is deeply concave, reminiscent of both the “morphotype 3” humerus associated with *Rhinochelys pulcriceps* by Evers et al.^2^ (p. 76, figure 22C), and various isolated *Rhinochelys*-like humeri reported from the mid-Cretaceous of Europe.^44^^,^^45^

Tong et al.^34^ discussed the distal forelimb elements of MIM F50, noting the elongate and anteriorly curving ulna (typical of protostegids^46^), anteriorly positioned radius (facilitating elbow hyperextension for “underwater flight”^3^), and extremely large proximal carpals, especially the ulnare, pisiform, and an associated supernumerary element that apparently occurs elsewhere in *Archelon*.^34^ Flattening and enlargement of the carpals, lengthening of the manual phalanges (with manual digit IV being longest), butt-joints between the manual digits, and retention of a distally “truncated” (Tong et al.*,*^34^ p. 129) third phalanx on manual digit V were all listed by Tong et al.*,*^34^ Evers et al.*,*^2^ and Joyce et al.^3^ as protostegid character states indicating rigid fore-flippers in *R. nammourensis.* Tong et al.^34^ (p. 129) emphasized that manual digit V was “situated well inside of the paddle,” as are digits I–IV (see Figures 1C and 1D), thus supporting our verification of a clawless fore-flipper (contra Tong et al.*,*^34^ p. 128). Tong et al.^34^ further interpreted all the manual digits as being rigid. However, the distal-most phalangeal articulations of MIM F50 are rounded and may have been partially mobile such as those of *Santanachelys*,^39^ possibly *Terlinguachelys*,^41^ and basal chelonioids, including *Toxochelys*.^47^

Bones from the right hindlimb, which Tong et al.^34^ observed only as impressions on the part slab, are preserved in articulation on the counterpart slab (Figures 1E and 1F), along with dispersed elements from the left hindlimb (Figure 1B). These are obversely visible on the part slab (Figure 1A), and include fragments of the pelvic girdle, femur, tibia, and fibula exposed in longitudinally split cross-section, and the tarsus and pes that mostly lie beneath the carapace. The femur (Figure 2F) is shorter than the humerus (a diagnostic trait of chelonioids^2^^,^^3^^,^^39^), but still large (49/57 mm in maximum femur/humerus length^34^) as in other protostegids,^41^ and the head is anterodorsally offset relative to the shaft such as those of *Terlinguachelys*^41^ and putative stem-cheloniids.^48^ We agree with Tong et al.^34^ in recognizing that the metatarsals and phalanges are much shorter than those of the forelimbs, and that pedal digits II–V all have three phalanges. Contrary to Tong et al.*,*^34^ the astragalus and calcaneum are sutured (not fused) and flattened (as in crown-lineage chelonioids^47^), and pedal digit V has two phalanges incorporating a pointed ungual. The metatarsal-phalangeal articulation surfaces form butt-joints, but the distal phalangeal joints are rounded, implying some flexibility. Pedal digits I–IV bear tapered unguals whose tips protrude just beyond the flipper edge as possible claws; whereas the terminal phalanx of pedal digit V is extremely reduced and entirely embedded in tissue.

### Analysis and implications

We used a Bayesian ancestral states analysis (see the STAR Methods) to infer the evolution of scales across a time-calibrated^2^ total-group chelonioid phylogeny.^2^^,^^3^^,^^4^ Our scaffold Bayesian tree (Figure 3) is broadly consistent with parsimony-based topologies produced elsewhere using the same dataset.^2^^,^^3^^,^^4^ However, we return crown-cheloniids and dermochelyids as sister lineages^3^^,^^50^ (albeit with limited node support), rather than disparately nested among stem-chelonioids,^2^^,^^3^ or with dermochelyids positioned as more basal relatives of protostegids (=Dermochelyoidea).^37^^,^^39^^,^^46^^,^^50^^,^^51^^,^^52^ Furthermore, our ancestral state estimations indicate that not only shell scutes,^11^ but also scaly limbs were probably archetypal for all chelonioids (probability % [P] = 0.93). Yet, the likelihood of scale loss increased (Table 1) in key clades as evinced by their soft tissue traces preserved in the fossil record. These include: (1) *Rhinochelys* (P = 0.83) and potentially other sea-going protostegids, such *Protostega* and *Archelon*, which apparently lacked scutes,^47^ implicating a trend toward body and shell scale loss across the Late Cretaceous (Cenomanian-to-Campanian).^49^ (2) Earliest Eocene crown-group chelonioids,^10^ but with modern cheloniids retaining scaly flippers (P = 0.85), possibly to prevent osmotic desiccation in salt water^3^ (although this hypothesis is counterintuitive to the repeated instances of scale loss evident in chelonioids^10^ and other marine reptiles^13^^,^^14^^,^^15^), or to provide traction on the seafloor while benthic feeding in coastal habitats.^10^^,^^14^ (3) The fully oceanic *Dermochelys*^10^ and closely related Paleocene–Eocene dermochelyids,^27^^,^^28^^,^^54^ whose divergence from scaly progenitors (P = 0.74) accompanied enhanced shell reduction (the carapace being almost entirely replaced by skin-covered osteoderms^55^), and a suite of morphological, physiological, and behavioral specializations for long-distance migration and deep diving.^11^

Finally, we suggest that differentiation of the elongate and apparently rigid fore-flippers^2^ from the compact and webbed hind-flippers in *Rhinochelys* could have been functionally coupled with scale loss to enhance “underwater flight.” This would have favored hydrodynamic smooth-skinned pectoral limbs for propulsion versus partially flexible pelvic limbs for maneuverability.^10^ Certainly, the proportionately large hind-flippers of *Rhinochelys* might still have facilitated slower-speed swimming involving synchronous lateral limb movements as posited for coastally restricted early cheloniids.^10^ Nevertheless, the neritic habitat^35^ and advanced aquatic traits of *Rhinochelys* (and related protostegids^2^^,^^37^^,^^46^^,^^47^^,^^50^^,^^53^) advocate multiple pelagic radiations within total-group Chelonioidea that integrated scale loss as an ecologically plastic adaptation, augmenting their opportunistic transitions toward oceanic lifestyles.

### Limitations of the study

While our research provides new insights into the antiquity of specialized pelagic adaptation in sea turtles, the extreme rarity and uniqueness of adequately preserved soft tissue fossils limit a broader understanding of integumentary character state distribution across all chelonioid taxa. Our analysis was therefore designed to accommodate this missing data, but we acknowledge that future fossil discoveries may (and should) refine both the phylogenetic resolution and ancestral conditions of some ingroup clades. Moreover, the fixed exhibition mounting and commercial preparation of MIM F50, although minimally obscuring and/or augmenting original anatomical features, did prevent an in-depth microscopic and spectroscopic analysis of the remnant soft tissues. Ideally, such shortcomings will be mitigated by the discovery of new remains, which we anticipate given that active quarrying is still underway at the Nammoura, Hâqel, and Hjoula Lagerstätten localities exposing the Sannine Formation.^35^

## Resource availability

### Lead contact

Further information and requests should be directed to and will be fulfilled by the lead contact, Benjamin P. Kear (benjamin.kear@nrm.se).

### Materials availability

MIM F50 is permanently housed and publicly accessible at the *MIM Mineral Museum Beirut*, Lebanon (https://www.mim.museum/). MIM is affiliated with the *Saint-Joseph University of Beirut*, Lebanon (https://www.usj.edu.lb/). Our phylogenetic dataset is provided as supplemental information. Our original photographic archive of MIM F50 has been uploaded onto the open-access *MorphoBank* (https://morphobank.org/) data repository under the permalink for Project 5666 (http://morphobank.org/permalink/?P5666).

### Data and code availability

• Data: All data used for analysis are publicly available with this article as of the date of publication.
• Code: This study does not report any original code.
• Additional information: Any additional information required to reanalyze the data reported herein is available from the lead contact upon request.

## Acknowledgments

Salim Eddé and staff (MIM), and Pierre Abi Saad (*Mémoire du Temps*) facilitated specimen access. *Expo Hakel* and the Nohra family contributed generous logistical support. Pollyanna von Knorring (*Swedish Museum of Natural History*) produced the artwork. Financial support was provided by a strategic partnership (*Verifiering för samverkan*) grant from 10.13039/501100007051Uppsala University to B.P.K., R.N., and M.B. B.P.K. acknowledges a 10.13039/501100004359Swedish Research Council grant (2020-3423). J.L. recognizes a 10.13039/501100004359Swedish Research Council grant (2020-3542). M.B. is funded by a Wallenberg Postdoctoral Scholarship (2022.0330). We thank the Editor and reviewers for constructive comments.

## Author contributions

B.P.K. conceived and designed the study, analyzed the data, interpreted the results, and wrote the article. R.N. and M.B. organized specimen access and logistics. J.L. and M.R. contributed specialist equipment and interpreted the results. All authors were involved in discussing the results and editing the article.

## Declaration of interests

The authors declare no competing interests.

## STAR★Methods

### Key resources table

**Table:** 

REAGENT or RESOURCE	SOURCE	IDENTIFIER
**Deposited data**		

Specimen repository	MIM Mineral Museum Beirut	https://www.mim.museum/
Image dataset	*MorphoBank*	http://morphobank.org/permalink/?P5666

**Software and algorithms**		

*MrBayes* v.3.2.1	Ronquist et al.^38^	https://nbisweden.github.io/MrBayes/download.html

### Experimental model and study participant details

Our study used empirical fossil data derived from first-hand inspections of original specimens (MIM F50) and published information from the literature.^5^^,^^6^^,^^7^^,^^8^^,^^10^^,^^16^^,^^17^^,^^56^ This primary data underpinned phylogenetic modelling of scale loss ancestry within total-group Chelonioidea^2^^,^^3^^,^^4^ and an outgroup sample of other testudinatan (= turtles and their stem relatives) clades.^56^^,^^57^ On-site photography and character scoring of MIM F50 was undertaken by B.P.K., M.B., and R.N. during two separate research visits from the 2^nd^–12^th^ May 2022 and 29^th^ January–4^th^ February 2023. These trips involved museum collection surveys at MIM, *Expo Hakel* (https://expo-hakel.com/), *Mémoire du Temps* (https://www.memoryoftime.com/), and field inspections of the Nammoura, Hâqel, and Hjoula Lagerstätten fossil localities of the Sannine Formation.^35^ All work was conducted in accordance with local regulations and requisite permissions from private and public authorities.

### Method details

#### Photography

MIM F50 was photographed using a *Nikon* D90 12.3 megapixel digital single-lens reflex (DSLR) camera equipped with a *Nikon* AF-S Micro-Nikkor 60 mm 1:2.8G macro lens for high-resolution close-up imaging of the preserved soft tissues. Because the part and counterpart slabs of MIM F50 are fixed to display mounts and thus only observable under exhibition spotlighting, we used both cross-polarization^58^ (CP) and ultraviolet (UV) photography (with direct spotlights turned off and the glass display case cover removed) for maximum contrast of the low-relief fossilized soft tissue remains.^10^ Our CP set-up employed a *Nikon* 62 mm circular polarizing lens filter and a camera-mounted LED light with trimmed polarizing film sheet taped to the light surface. For UV photography, we used both tripod-mounted and mobile hand-held UV lamps (cut-off at ∼365 nm) for optimal illumination of the fossil.

#### Phylogenetic dataset selection and ancestral states analysis

Although the topological placement^59^ and classification^57^ of some protostegid taxa within Chelonioidea has been questioned, phylogenetic resolution of Protostegidae *sensu lato*^47^^,^^49^ with total-group Chelonioidea is consistent and well supported.^2^^,^^3^^,^^4^^,^^37^^,^^39^^,^^46^^,^^47^^,^^50^^,^^52^^,^^53^^,^^60^ We therefore selected the ingroup clade taxonomy and dataset of Evers et al.^2^ as modified by Joyce et al.^3^ and Menon et al.^4^ because this provides the most recent published phylogeny of total-group Chelonioidea, and was also specifically designed to analyze the relationships of *Rhinochelys*.^2^ We followed the original parameters of Evers et al.*,*^2^ who treated all multistate characters as unordered and designated *Proganochelys quenstedtii* as the most distant outgroup taxon. We then scored a novel character numbered ‘357’ that coded the presence of limb scales (state ‘0’) versus limb scale loss (state ‘1’) across 30 extant testudine^3^^,^^10^^,^^11^^,^^56^ (see a complete species list in Data S1 [phylogenetic dataset file], related to Table 1) and four extinct species-level taxa collectively representing 16 separate family-level clades/stem lineages.^56^^,^^57^ Fossils included: the basally divergent testudinatans^59^ *P. quenstedtii*^5^^,^^6^^,^^8^ and *Meiolania platyceps*,^16^^,^^17^ both of which preserve probable scute-covered limb osteoderms; the scaly limbed thalassochelydian *Thalassemys bruntrutana*^3^; and *Rhinochelys nammourensis*,^33^ whose soft-skinned flippers reveal scale loss among protostegids. Our NEXUS file comprising 97 taxa and 357 characters is provided in Data S1 ([phylogenetic dataset file], related to Figure 3) and has also been uploaded onto the open-access *MorphoBank* Project 5666 (http://morphobank.org/permalink/?P5666).

We ran an initial analysis in *MrBayes* v.3.2.1^38^ to generate our Bayesian consensus tree and check node support for major clades (Figure 3). Settings included the default *Mkv* model for morphology^61^ with a ‘gamma’ rates variation parameter and ‘variable’ coding for heterogeneity. Two simultaneous runs and eight Markov Chains were applied for 5,000,000 generations with default settings and the burn-in fraction set at 0.25.

We then inferred ancestral state probabilities for a data partition of character 357 versus characters 1–356 across a series of enforced topological constraints corresponding to the best supported (PP >0.9) target clade nodes (Table 1). (1) Total-group Chelonioidea = *Toxochelys* sp. + *Nichollsemys baieri* + Protostegidae + Ctenochelyidae + *Argillochelys cuneiceps + Eochelone brabantica + Erquelinnesia gosseleti + Oligochelone rupelensis + Procolpochelys charlestonensis + Puppigerus camperi* + crown-group Chelonioidea. (2) Protostgidae + crown-group Chelonioidea = Protostegidae + Ctenochelyidae + *A. cuneiceps + E. brabantica + E. gosseleti + O. rupelensis + P. charlestonensis + P. camperi* + crown-group Chelonioidea. (3) Protostegidae = *Archelon ischyros + Bouliachelys suteri + Calcarichelys gemma + Chelosphargis advena + Desmatochelys lowii + Desmatochelys padillai + Notochelone costata + Ocepechelon bouyai + Protostega gigas + Rhinochelys pulchriceps + R. nammourensis + Santanachelys gaffneyi*. (4) *Rhinochelys* = *R. pulchriceps + R. nammourensis*. (5) Ctenochelyidae + crown-group Chelonioidea = Ctenochelyidae + *A. cuneiceps + E. brabantica + E. gosseleti + O. rupelensis + P. charlestonensis + P. camperi* + crown-group Chelonioidea. (6) Crown-group Dermochelyidae = *Dermochelys coriacea + Eosphargis breineri*. (7) Crown-group Cheloniidae = *Caretta caretta + Chelonia mydas + Eretmochelys imbricata + Lepidochelys kempii + Lepidochelys olivacea + Natator depressus*. These analyses each used two simultaneous runs and 1,000,000 generations with default settings.

### Quantification and statistical analysis

Our quantification and statistical analysis of the data included a series of Bayesian phylogenetic and ancestral states approaches that are explained in the method details.

## Supplemental information

Data S1. SD1 Phylogenetic dataset, related to the STAR Methods
